# Role of the IL-2 inducible tyrosine kinase ITK and its inhibitors in disease pathogenesis

**DOI:** 10.1007/s00109-020-01958-z

**Published:** 2020-08-18

**Authors:** Kristina S. Lechner, Markus F. Neurath, Benno Weigmann

**Affiliations:** 1 Department of Medicine 1, Kussmaul Campus for Medical Research, University of Erlangen-Nürnberg, Hartmannstr.14, 91052 Erlangen, Germany; 2 Deutsches Zentrum Immuntherapie (DZI), Ulmenweg 18, 91054 Erlangen, Germany; 3 Ludwig Demling Endoscopy Center of Excellence, Ulmenweg 18, 91054 Erlangen, Germany; 4 Medical Immunology Campus Erlangen, Medical Clinic 1, Friedrich-Alexander University Erlangen-Nürnberg, 91052 Erlangen, Germany

**Keywords:** ITK, Autoimmune diseases, Cancer, Inhibitor

## Abstract

ITK (IL-2-inducible tyrosine kinase) belongs to the Tec family kinases and is mainly expressed in T cells. It is involved in TCR signalling events driving processes like T cell development as well as Th2, Th9 and Th17 responses thereby controlling the expression of pro-inflammatory cytokines. Studies have shown that ITK is involved in the pathogenesis of autoimmune diseases as well as in carcinogenesis. The loss of ITK or its activity either by mutation or by the use of inhibitors led to a beneficial outcome in experimental models of asthma, inflammatory bowel disease and multiple sclerosis among others. In humans, biallelic mutations in the ITK gene locus result in a monogenetic disorder leading to T cell dysfunction; in consequence, mainly EBV infections can lead to severe immune dysregulation evident by lymphoproliferation, lymphoma and hemophagocytic lymphohistiocytosis. Furthermore, patients who suffer from angioimmunoblastic T cell lymphoma have been found to express significantly more ITK. These findings put ITK in the strong focus as a target for drug development.

## Introduction

ITK was first reported in the late 1980s and early 1990s when it was first cloned [1–5]. The name of ITK originates from the finding that its expression was induced by the administration of IL-2 in IL-2-deprived cells [1]. This protein is also named Emt [2, 5] and Tsk [3] and belongs to the Tec family of non-receptor tyrosine kinases (TFK) [6]. Besides ITK, the TFK consists of four other members namely Btk (Bruton’s tyrosine kinase), Tec, Rlk (also known as Txk) and Bmx [6]. While Btk is mainly expressed in B lymphocytes [7], ITK, alongside with Rlk and Tec, is preferentially expressed in T lymphocytes whereas ITK shows the highest level of expression [1–3, 8]. Apart from that, ITK is also expressed in mast cells [9], natural killer cells [10] and invariant natural killer T cells (iNKT) [11]. Since up to date only ITK has been found to have a defined function in the T cell lineage, this kinase is considered to be the predominant Tec kinase in T cells [9].

The domain structure of ITK and members of the TFK in general reveals unique features and also features shared with kinases belonging to the Src family kinases. ITK consists of an N-terminal pleckstrin-homology domain (PH) which is followed by a proline-rich Tec homology region (TH) and the Src homology 2 (SH2) and 3 (SH3) domain. On the carboxy-terminal end lies the specific kinase catalytic domain [8, 9]. However, the TFK lack the N-terminal myristoylation signal and the C-terminal regulatory tyrosine residue found in Src family kinases [1]. The PH domain is important for recruitment to the plasma membrane [12, 13]. The SH2 domain regulates protein-protein interactions while the SH3 domain binds to the proline-rich motif in the TH domain which leads to auto-inhibition of ITK [14–17].

**Fig. 1:**
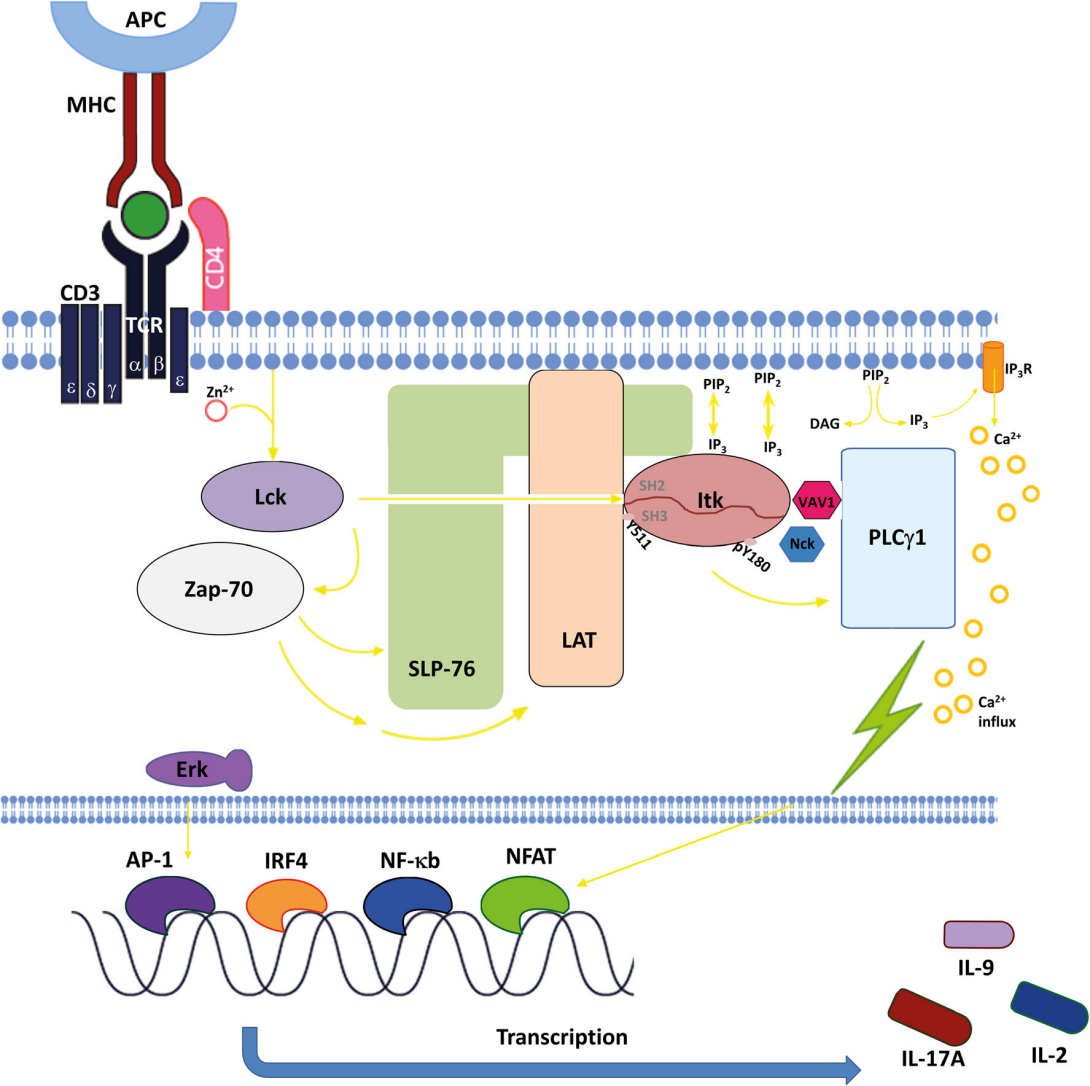
Interleukin-2-inducible T cell kinase plays a central role in the signal transduction of the T cell receptor. By binding of the MHC to the TCR, the kinase Lck (lymphocyte-specific protein tyrosine kinase) becomes activated with the help of Zn^2+^. Lck binds to protein Zap-70 (zeta-chain-associated protein kinase 70) which leads to the phosphorylation of LAT (linker for activation of T cells) and SLP-76 (SRC-homology-2-domain-containing leukocyte protein of 76 kDa). Upon binding of ITK to PIP_3_ (phosphatidylinositol-3,4,5-triphosphate) via its PH domain, ITK is recruited to the LAT/SLP-76-signalling complex. ITK interacts with SLP-76 and LAT at its SH2 and SH3 domains and gets phosphorylated on the tyrosine residue Y511 and Y180. The LAT/SLP-76 complex can also function as an accumulation platform for complex proteins like VAV1 (vav guanine nucleotide exchange factor 1) and Nck (non-catalytic region of tyrosine kinase). ITK becomes activated which results in the phosphorylation of PLCγ1 (phospholipase Cγ1) and the generation of IP_3_ (inositol-1,4,5-triphosphate) and DAG (diacylglycerol), which activates the PKC (protein kinase C). Finally, a Ca^2+^ influx within the cell can be observed. Through this signalling pathway, ITK can control the nuclear translocation of transcription factors AP-1 (activator protein 1) via Erk (extracellular-signal-regulated kinase), NFAT (nuclear factor of activated T cells), IRF4 (Interferon Regulatory Factor 4) and NF-κb (nuclear factor ‘kappa-light-chain-enhancer’ of activated B-cells) as well as the subsequent expression of various genes, e.g., IL-2, IL-9 and IL-17A

The T cell receptor (TCR) can recognize MHC complexes on antigen-presenting cells (APC). The binding of these complexes leads to an activation of the Src kinase Lck which phosphorylates the CD3 immunoreceptor tyrosine activation motifs (ITAMs). This leads to the binding of Zap-70 which is in turn phosphorylated by Lck and therefore activated. Zap-70 phosphorylates the adaptors LAT and SLP-76 which serve as a platform for recruitment of ITK, Vav1 and Nck to form a signalling complex. Following the co-stimulation of the T cells with CD28, PI3K is activated and PIP_3_, the product of PI3K, is accumulated. ITK can bind to PIP_3_ via its PH domain which results in the recruitment of ITK to the signalling complex at the cell membrane. There, ITK interacts with SLP-76 and LAT via its SH2 and SH3 domains. Consequently, ITK gets phosphorylated on the tyrosine residue 511 (Y511) by Lck which in turn leads to the autophosphorylation step of ITK on Y180 which lies in its SH3 domain. Besides that, ITK also interacts with the downstream molecule PLCγ1 and activates it by phosphorylating this molecule. This leads to the hydrolyzation of PIP_2_ into IP_3_ and DAG which serve as second messengers. Further downstream effects occurring after the activation of the TCR signalling pathway include Ca^2+^ influx; the activation and translocation of transcription factors like AP-1, IRF4 and NFAT into the nucleus; the induction of transcription; and the production and release of cytokines like IL-2, IL-9 and IL-17A [9, 18]. The complete signalling pathway is demonstrated in Fig. 1.

Former studies have shown that the loss of ITK in ITK-deficient mice has a strong effect on the production and accumulation of cytokines typical for Th2 cells [19–21]. ITKKO mice that have been infected with the parasites *Leishmania major* or *Nippostrongylus brasiliensis* were not able to generate a protective Th2 response necessary for these infections to be cleared. Instead, an upregulation of Th1 cytokines could be detected [19]. However, the loss of ITK alone does not have a strong effect on the development of Th1-related cytokines since in Th1 cells also Rlk is expressed and can therefore compensate for the loss of ITK [8, 9]. Moreover, the loss of ITK also affects Th17 cells, a T cell subset important for antimicrobial activity especially in the gastrointestinal tract [9]. It was found that ITK influences the production of Th17 cytokines as well. Gomez-Rodriguez et al. demonstrated that under in vitro conditions, the depletion of ITK leads to less expression of IL-17A. This effect is independent from the altered thymic development found in ITKKO mice since re-expression of ITK in activated ITKKO CD4+ cells could rescue the IL-17A expression. Nevertheless, the mRNA level for the genes of the key transcription factor for Th17 cells, RORγT, and other cytokines linked to Th17 cells like IL-17F, IL-21 and IL-22 remained unaffected by the loss of ITK [22]. The same group also demonstrated that ITK-deficient CD4+ T cells that were polarized under Th9 conditions were not able to produce IL-9 compared with WT CD4+ T cells. Besides, they showed that the ITK-deficient cells could not express IRF4 which is an important transcription factor for Th9 differentiation. On the other hand, the administration of IL-2 could rescue IL-9 and IRF4 expression highlighting the fact that ITK is necessary for Th9 differentiation [18]. The role of ITK in T regulatory cells (Tregs) is slightly contradictory. On the one hand, it has been shown that ITKKO mice show an upregulated proportion of CD4+FoxP3+ double-positive cells [23]. On the other hand, Huang et al. demonstrated that although ITK represses the development of Tregs, it is still necessary for the proper suppressive function of Tregs [24]. Type 1 regulatory T cells (Tr1) only show little to nearly no expression of FoxP3. However, they produce high levels of IL-10 and can suppress effector cell responses [25, 26]. Huang et al. showed that ITK is required for Tr1 cell development as well as for their suppressive function in both human and mice. ITK regulates the function of Tr1 cells via the IRF4/Ras pathway. The loss of ITK leads to less IRF4 expression and therefore a reduced function of Tr1 cells. However, this phenotype could be reverted by re-expression of IRF4 [27]. Furthermore, it was shown that ITK is needed for ILC2 survival too. In the intestinal lamina propria of mice lacking ITK, an increased loss of ILC2 alongside with less tissue integrity occurred. However, this phenotype could be reverted by administration of IL-2-complexes into ITKKO mice [28]. Lastly, ITK is also responsible for the calcium signalling after TCR activation and thereby controls the activity and translocation of transcription factors dependent on calcium influx like NFAT and NFκB [19, 29–31].

Various studies have shown that ITK-depleted mice have reduced numbers of mature thymocytes in spleen and lymph nodes and in concordance with that also decreased proliferative responses after TCR cross-linking. Furthermore, it was demonstrated that these mice also have a lower CD4+:CD8+ ratio and defects on thymic selection as well as T helper cell development [19, 29, 32]. By implication, one can fully suggest that therefore ITK plays a very pivotal role in T cell development and differentiation.

By and large, ITK plays a central role in the TCR signalling pathway in a great variety of cell subsets, controlling the expression of calcium-dependent transcription factors like NFAT and NFκB and the expression of pro-inflammatory cytokines from Th2, Th9 and Th17 cells as well as mast cells [33]. In summary, ITK plays a key role in inflammatory processes [34].

### The role of ITK in inflammatory processes

**Fig. 2:**
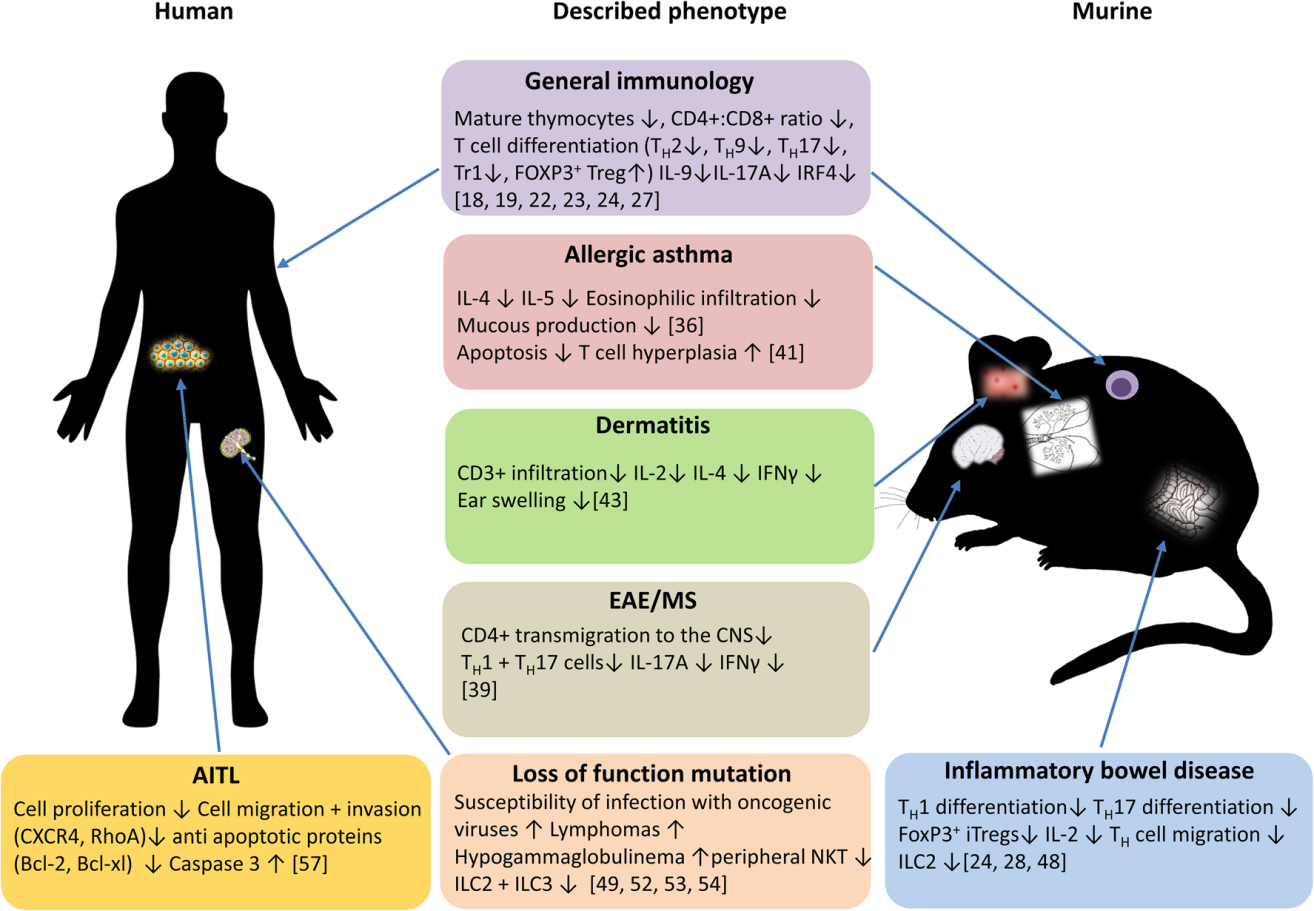
Schematic comparison between the numerous phenotypes in murine and human immune systems caused by ITK deficiency. The loss of ITK has numerous effects on different malignancies in both mice and humans. In general, ITK deficiency results in less mature thymocytes in spleen and lymph nodes, defects in T cell differentiation and a decrease in IL-9, IL-17A and IRF4 expression. In the lung, the role of ITK is contradictory, as there are studies showing that ITK deficiency leads to less cell infiltration and less mucous production whereas other studies demonstrated that the loss has no beneficial effect and instead leading to T cell hyperplasia. Mice studies have shown that the loss of ITK results in a better outcome in experimental dermatitis models, with less T cell infiltration, cytokine secretion and ear swelling. In experimental mice models of multiple sclerosis, ITK deficiency leads to less CD4+ cell transmigration into the CNS as well as less IFNγ and IL-17A secretion. Also, in the gut, studies with experimental murine colitis models have shown that the loss of ITK leads to less Th1 and Th17 differentiation alongside with less expression of FoxP3 and IL-2. A loss of function mutation as described in humans leads to a higher susceptibility to viral infections with oncogenic viruses, development of lymphomas and hypogammaglobulinemia. On cellular basis, a decrease in peripheral NKT, ILC2 and ILC3 was detected. Lastly, in human T cell leukaemia and lymphoma cell lines, it was demonstrated that the loss of ITK leads to less cell proliferation, cell migration and cell invasion together with a higher expression of pro-apoptotic genes

Numerous studies could show that ITK is involved in various inflammatory diseases. An overview of the numerous ITK-caused phenotypes as compared between murine and human immune systems is shown in Fig. 2.

Among those are allergic asthma [35–38], atopic dermatitis, aplastic anaemia and peripheral T cell lymphomas [34]. Moreover, Kannan and colleagues demonstrated that ITK is also deeply involved in promoting neuroinflammation [39].

In the case of allergic asthma, the role of ITK during inflammation seems to be dichotomous as various studies suggest. On the one hand, several papers have shown that the loss of ITK leads to a reduced airway inflammation due to less infiltration of Th2 cells into the pulmonary lung tissue after treatment with allergens [36, 40]. On the other hand, there are also several studies that show the exact opposite. Sun et al. discovered that although ITKKO mice are resistant to airway inflammation, the use of a specific ITK-selective inhibitor failed to reduce inflammation after re-challenging the mice with allergens. The T cell number as well as the Th2 cytokines increased in the airways and therefore they suggested that the blocking of ITK in human patients with asthma would possibly not have any therapeutic effect [41].

In patients suffering from atopic dermatitis, elevated levels of ITK were found in peripheral blood T cells [42]. Again, ITKKO mice have a beneficial outcome in models of acute contact hypersensitivity reactions and show reduced inflammation [43]. Treatment with a specific ITK inhibitor and using siRNA against ITK could also reduce the inflammatory symptoms in mice. In human cells, both approaches of blocking ITK led to a reduced IFNγ and IL-2 production alongside with less proliferation [43]. However, the molecular mechanism by which ITK drives the inflammatory responses in atopic dermatitis is still unknown and needs to be further elucidated.

Solomou et al. investigated the role of ITK in patients with aplastic anaemia. This disease is characterized by an empty bone marrow and in most cases T cells are the main factor of destroying haematopoietic cells [44, 45]. Since it is already known that ITK is engaged in the activation of T-bet [46], and since they found that patients with this disease showed high levels of T-bet, they suggested that there could be a connection between T-bet and ITK in the inflammatory responses of aplastic anaemia. In this study, they could point out that in patients, the increased T-bet protein level correlates with the protein level of ITK [47].

There is also some evidence that ITK might have a detrimental role in inflammatory bowel disease. The two main diseases impacting the gastrointestinal tract are Crohn’s disease and ulcerative colitis. While Crohn’s disease can occur in any part of the gastrointestinal tract, from the mouth to the anus, ulcerative colitis is restricted to the colon. However, in most cases, Crohn’s disease affects the ileum and the beginning of the colon whereas ulcerative colitis starts in the rectum and moves towards the ileum. Both of them share symptoms like abdominal pain, diarrhoea and rectal bleeding. In a study performed by Cho et al., it was found that an inhibitor of ITK and Rlk (PRN 694) can prevent Th1 differentiation, and therefore, colitis development. This leads to the reverse conclusion that ITK might also play an important role in the development of inflammatory bowel disease. They demonstrated that after treatment with the inhibitor PRN 694 in a transfer colitis model, the mice showed a smaller proportion of CD4+ cells in the lamina propria (LP) as well as in the intestinal epithelium. Furthermore, there were less detectable IFNγ secreting CD4+ cells in the LP, mesenteric lymph nodes and intraepithelial lymphocytes [48]. Although it has already been pointed out before that ITKKO mice show upregulated expression of regulatory T cells in adoptive colitis model leading to a protective phenotype due to an increased expression of FoxP3 [23], Cho et al. could additionally reveal that the inhibition of ITK led to an decreased expression of CD4+ FoxP3+ double-positive cells [48]. This finding is contradictory to the study from Huang et al. which demonstrated that ITKKO mice had an increased amount of Tregs. Therefore, they suggested that the regulation of the Treg differentiation is dependent on the kinase activity of ITK. However, the same study also revealed that ITK is required for the suppressive function of Tregs in inflammatory diseases: In Rag1−/−, reconstituted with naïve CD4+ cells, a co-transfer of CD4+CD25+ cells, derived from ITKKO mice, could not prevent the development of colitis. In contrast, CD4+CD25+ cells from WT mice inhibited the development of inflammation. [24].

Interestingly enough, ITK also has a pivotal role in neuroinflammatory diseases as shown by Kannan et al. They found out that ITK encourages experimental autoimmune encephalomyelitis (EAE), which serves as a model for multiple sclerosis (MS). In reverse, they showed that the depletion of ITK results in a diminished disease severity in mice with less transmigration of CD4+ cells into the central nervous system and across the brain endothelial barrier as well as reduced secretion of Th1 and Th17 effector cytokines. In conclusion, it was suggested that ITK signalling in CD4+ cells features prominently in neuroinflammatory diseases [39].

On the whole, these findings show that ITK has a multifunctional and an extremely central role in various severe diseases. That ITK can potentially play a strong role for therapeutics to treat different kinds of auto- and neuroinflammatory diseases is evident from all these studies.

### The role of ITK in oncogenesis

Besides its decisive participation in inflammatory processes, various studies could also demonstrate that ITK is involved in oncogenesis. Here especially, observations concerning the occurrence of non-Hodgkin and Hodgkin lymphomas were made in recent years.

A study carried out by Huck et al. demonstrated that the loss of ITK function due to mutation in patients can lead to an EBV-associated lymphoproliferation [49]. Epstein-Barr-Virus (EBV) belongs to the family of gammaherpesvirus and about 90% of the world’s population acquire this virus during childhood where it mostly persists in a latent state. In patients who suffer from immunodeficiencies, for example due to defects in T cell signalling, the infection with EBV can lead to severe immune dysregulations. The infections can lead to Hodgkin and non-Hodgkin lymphoma, mononucleosis, lymphoproliferative disease, dysgammaglobulinemia and hemophagocytic lymphohistiocytosis [50, 51]. In 2009, the first two patients carrying a homozygous missense mutation in the ITK gene at position C1085T leading to an exchange of arginine by tryptophan at position 335 (R335W) were discovered by the group of Arndt Borkhardt. The residue 335 is located in the SH2 domain of ITK and the substitution of the amino acids leads to a protein destabilization of SH2. Both patients were diagnosed with an EBV infection and developed Hodgkin lymphoma [49]. Since then, 17 more patients who suffer from genetic mutations of ITK have been found. In most of these patients, a decreased number of CD45RA+ CD4+ T cells as well as peripheral NKT cells, characterized as CD3+, TCR Vβ11+ and TCR Vα24+, were found [52]. This finding matches with phenotypes seen in ITK-deficient mice showing dysfunctional NKT cells which have a reduced survival rate in the periphery [11]. Furthermore, in most of these patients, a progressive hypogammaglobulinemia was seen. This is in concordance with other EBV-prone disorders [53]. Interestingly, in a study by Eken et al., reduced numbers of ILC2 and ILC3 together with Th17 cells were observed in an ITK-deficient patient [54].

Recently, another case was presented in two siblings where the mutation of the ITK gene in combination with an infection with the cutaneous human papillomavirus (HPV) type 5 and 8 resulted in epidermodysplasia verruciformis (EV) and Hodgkin lymphoma. EV is characterized by cutaneous flat warts which can progress into squamous cell carcinomas later in life. The mutation of ITK was a 3-bp deletion and occurred on exon 4 in the BTK-type zinc finger domain which contains the conserved histidine 121. This histidine is important for the stability of the BTK motif plus the interaction between Zn^2+^ ions and cysteine. Susceptibility of HPV infection due to loss of ITK has not been reported before and is a complete novel finding [55].

A possible explanation for the predisposition for infections with oncogenic viruses and the subsequent development of lymphomas in patients with ITK deficiency is provided by Kapnick et al. The group demonstrated in murine cytolytic T lymphocytes (CTLs) that ITK deficiency affects both the early differentiation and expansion of CTLs and the late stages of cytolytic activity. Furthermore, they showed that ITK is needed for combating target cells and that ITK-deficient CTLs have defects in degranulation leading to a lack of cytotoxicity [56]. Moreover, Linka et al. analysed the calcium influx in patients with mutations of ITK affecting different domain structures. They revealed that those patients had a remarkably reduced calcium response after TCR stimulation thereby impeding T cell immune responses [53]. Overall, these findings could supply a possible answer to the question why patients with ITK-deficiency have a higher susceptibility to viral infections as well as a decreased viral clearance. However, these observations are in contrast to the previous finding, namely, that the loss of ITK leads to a beneficial outcome in murine models of autoimmune diseases especially affecting Th2 and Th17 cells.

In the cases described above, a loss of function mutation of ITK led to the occurrence of lymphomas after viral infections. This is due to the fact that these patients have a T cell dysfunction leading to less immune surveillance. Upon viral infections and because of the dysregulated T cells, less tumour surveillance will take place finally resulting in the development of lymphomas [52]. Therefore, the occurrence of these malignancies is a consequence of ITK deficiency. Besides that, there are also several well-known cases where an aberrant activation of ITK in already persisting tumours was found.

In the angioimmunoblastic T cell lymphoma (AITL) which is a subtype of non-Hodgkin peripheral T cell lymphomas (PTCLs) in general, studies have shown that the activation of the TCR signalling pathway might play a pivotal role in its pathogenesis [57]. AITL accounts for about 20% of PTLCs [58, 59] and patients have a very poor clinical outcome with only a 25 to 30% 5-year overall survival rate [58, 60]. Symptoms include lymphadenopathy, hepatosplenomegaly, pruritic skin rash, anaemia and hypergammaglobulinemia [58, 61]. Previously, it has been shown that in cells from AITL patients, ITK is a highly expressed marker [62] and that a SYK-ITK fusion protein drives the oncogenesis by activating the phosphorylation of TCR-proximal proteins independently of an antigen-binding [63]. In over 70% of the AITL patients investigated in the study by Liu et al., ITK was highly phosphorylated in cells of the tumour tissue. Moreover, of the patients which were treated with first line therapy, those who expressed phosphorylated ITK had a lower complete response than those negative for ITK phosphorylation. In addition, patients whose cells were positive for ITK-phospho expression had a lesser progression-free survival as well as an overall survival compared with those patients whose cells were negative for ITK-phospho expression. Furthermore, they showed that via ITK inhibition, the invasion and migration of malignant T cells could be decreased most likely due to downregulation of CXCR4, RhoA and FAK. Lastly, it was demonstrated that the blocking of ITK leads to an increased apoptotic phenotype in malignant T cells by increasing the expression of pro-apoptotic proteins like Bak while decreasing the expression of anti-apoptotic proteins like Bcl-2, Bcl-xl and Mcl-1 [57].

Finally, one study suggests that ITK might also play an important role in tumour proliferation in melanoma cells. This was a surprising finding regarding the fact that ITK expression is normally restricted to immune cell subsets as mentioned above. Carson et al. found out that the CpG islands of ITK are hypomethylated in melanoma compared with nevi and therefore primary and malignant melanomas show a higher expression of ITK compared with benign nevi. They could however not see a significant expression of ITK in the most common tumour-infiltrating immune cell lines. Overall, the role of ITK in melanomas needs to be further elucidated [64].

The above findings demonstrate that the mutation of ITK as a monogenetic disorder, which negatively influences the function of T cells, can lead to viral susceptibility, especially EBV and HPV, and thus to the development of lymphomas. Although in these cases the mutation of ITK led to the occurrence of lymphomas, there are also cases where an upregulation and activation of ITK occurs in already present tumours like in AITL or melanoma cells. However, in these latter cases, ITK activation in tumour cells is not linked to a predisposition for viral infections.

Despite the questionable effect of ITK inhibitors on lymphomas having been developed after viral infections, ITK is an important regulator of T cell development and activity and its inhibition could have beneficial effects on a variety of diseases, especially on autoimmune diseases as shown above. Therefore, a lot of emphasis has been put on finding and developing functional ITK inhibitors.

### ITK inhibitors

ITK plays a central role in regulating signalling pathways that can lead to inflammatory responses. For example, the production of cytokines from Th2 cells and mast cells [34] as well as the maturation of iNKT and the expression pro-apoptotic genes [65] are affected by an aberrant ITK expression. Moreover, ITK influences the activation of transcriptions factors like NFAT, NFκB, AP-1 and their regarding downstream events through the Ca^2+^ influx [34]. Therefore, it is logical to make ITK a key target for designing new inhibitors to tackle especially T cell–associated diseases. Up to date, numerous ITK inhibitors have been developed, each of which has been shown to be more or less sufficient in blocking ITK activity. In the scope of this review, we want to focus on the most prominent one as well as on very promising and newly designed ITK inhibitors.

Since ITK consists of distinct domains as described above, there are different approaches to block ITK. It is possible to either attack the ATP-binding site in the kinase domain thereby blocking the kinase activity [66–69] or it is also possible to target the PH domain of ITK to prevent recruitment to the plasma membrane [13]. Obviously, also the SH2 and SH3 domains could be suitable targets for the inhibition of the ITK function but since these domains have key roles in regulating not only ITK but also signalling events that are independent of ITK, more work has to be done before trying to block them [34].

The B cell homologue BTK has already been long in the focus of research compared with ITK; therefore, a variety of BTK inhibitors have been developed like LFM-A13, dasatinib or ibrutinib [70]. However, since ITK is getting more attention because of its involvement in a variety of inflammatory responses, inhibitors are getting developed. In the early 2000s, the first ITK-specific inhibitors were developed and comprised aminothiazoles (Bristol-Meyer-Squibb), aminobenzimidazoles (Boehringer Ingelheim), indoles (Sanofi-Aventis) and pyridones (Vertex) to only name a few [71] (Table 1). In recent years, it has been shown that ibrutinib is not only specific for BTK alone but also blocks ITK function due to their close homology [72, 73]. Initially, it was thought to be used against B cell malignancies as preclinical and clinical trials have shown that ibrutinib has a beneficial effect in patients with chronic lymphatic leukaemia [74], multiple myeloma [75] and mantle cell lymphoma [76]. However, in experimental models, ibrutinib also shows strong effects against T cell–associated inflammatory diseases like rheumatoid arthritis [77, 78] and asthma [79]. Recently, there has also been a pilot clinical trial of ibrutinib as an inhibitor of ITK in patients with relapsed T cell lymphoma [80].

**Table 1:** List of ITK inhibitors and their potential therapeutic application area

Name/Code	Company	Therapeutic use	Effect	References
BMS-509744 (aminothiazole)	Bristol-Meyer-Squibb	n.d. → No clinical trial	Prevented lung inflammation in experimental mouse model	[66]
Compound 1 and 2 (aminobenzimidazoles)	Boehringer-Ingelheim	n.d.	First inhibitor reported to have an in vivo oral function	[71]
Indole analog 9 (indole)	Sanofi-Aventis	No potential therapeutic use	Inhibited IL-4 secretion in mouse splenocytes with an IC_50_ of 11.3 nM	[71]
Compound 10 (pyridone)	Vertex	Potential therapeutic use for asthma and acute rhinitis	Inhibits RLK and ITK	[71]
Ibrutinib/PCI-32765	AbbVie	Approved in the EU for patients with CLL and B cell lymphomas. Potential therapeutic use for asthma, rheumatoid arthritis and T cell lymphoma	Inhibits BTK and ITK	[72, 77–80]
PRN694		Experimental model of psoriasis Experimental model of colitis	Inhibits Rlk and ITK	[48, 82]
Compound 9		Anti-proliferative effect in T cell leukaemia- and lymphoma cell lines in experimental models	Inhibits ITK	[83]
PF-06651600	Pfizer	Potential therapeutic use for rheumatoid arthritis and inflammatory bowel disease	Inhibits JAK3 and the Tec kinases + inhibits cytolytic function of CD8+ cells and NK cells	[84]
J-13		n.d.	Inhibits ITK by binding outside the ATP pocket	[85]
ECPIRM		Anti-proliferative effect in CTCL cells in experimental models	Inhibits ITK + inhibited tumour growth in Hut78-xenografted mice	[86]
CPI-818	Corvus Pharmaceuticals	Clinical phase 1/1B for the treatment of T cell lymphomas	Needs to be evaluated	

In 2014, the group of Jason A. Dubovsky discovered a small, irreversible inhibitor of ITK and Rlk called PRN694. This inhibitor binds the C442 residue in ITK and forms a covalent bond with it. PRN694 was checked for efficiency and function and it was found that this inhibitor blocks TCR-proximal signalling, Ca^2+^ influx and TCR-induced cell activation. Furthermore, it also diminished cytokine secretion from Th2 and Th17 cells due to selective inhibition of ITK and Rlk kinase activity [81]. One year later, it was published that Cho et al. administrated PRN 694 in an adoptive T cell transfer model of colitis and detected that this treatment reduced Th1 responses, stopped T cell infiltration into the colon, decreased IL-2 production of transferred CD4+ cells and reduced overall disease progression [48]. Recently, PRN 694 was also shown to have a beneficial effect in experimental mouse models of psoriasis [82].

Furthermore, Tang et al. described a covalent inhibitor of ITK based on the 7H-pyrrolo[2,3-d]pyrimidine scaffold named compound 9. This inhibitor docks into the ATP-binding pocket of ITK and thereby inhibits its activation. Moreover, compound 9 showed a 250-fold greater selectivity for ITK over BTK as well as successful inhibition of phosphorylation of PLCγ1. Therefore, compound 9 could be a promising new inhibitor to target ITK [83].

PF-06651600 is an inhibitor that interacts with the cysteine residue Cys-909 of JAK3. However, this residue is also found in the five members of the Tec family kinases at the equivalent position and therefore they can also be blocked by this specific inhibitor. In the study carried out by Xu et al., it was shown that PF-06651600 was able to block ITK on protein level in a dose-dependent manner in both Jurkat cells and in CD3/CD28/CD2-stimulated human CD4+ cells. Furthermore, they demonstrated that the inhibition of function of CD8+ cytotoxic T cells and NK cells occurred through the blockage of the Tec kinases with exception of BTK. They suggested that this inhibitor could lead to beneficial outcomes in an array of different diseases like vitiligo, inflammatory bowel disease or rheumatoid arthritis. However, one has to keep in mind that this inhibitor is not solely specific for ITK [84].

In addition, Hantani et al. discovered a new ITK inhibitor that does not bind to the ATP-binding pocket. The inhibitor J-13 showed great selectivity against a broad kinase panel and the group suggested that it might be a specific inhibitor for ITK. However, more studies have to be carried out to investigate the structure of ITK in complex with J-13 as well as to understand its binding mechanism [85].

ECPIRM is a retinoid derivant that exhibits anti-proliferative effects on CTCL (cutaneous T cell lymphoma) cells. Li et al. suggested that ITK might be the main target of ECPIRM since they found that ECPIRM interacts with ITK by binding to its hydrophobic active pocket. The hydrogen bonds formed by ITK are located at Ile^369^, Gly^441^ and Cys^442^ [86]. While Ile^369^ lies in the N-terminal lobe, both Gly^441^ and Cys^442^ are part of the C-terminal lobe. Both lobes are connected via a hinge region. Together, this pocket comprises the ATP-binding side [87]. Furthermore, they showed that the administration of ECPIRM to the human CTCL cell line Hut78 significantly reduced the protein level of ITK. Subsequently, this blockage of ITK led to a reduced tumour growth in Hut78-xenografted nude mice [86].

Very recently, an abstract was published introducing a novel selective inhibitor for ITK called CPI-818. In in vitro experiments, CPI-818 administration inhibited PLCγ1 phosphorylation and IL-2 secretion in Jurkat cells. Furthermore, the effect of CPI-818 was evaluated in vivo on dogs with spontaneously occurring T cell lymphomas. Here, this inhibitor was able to block ITK which led to an anti-tumour activity. The conclusion is that this inhibitor might also have a beneficial effect in human patients with T cell malignancies [88]. Currently, as of July 2020, this inhibitor is in phase 1/1B clinical trial for T cell lymphomas led by Corvus Pharmaceuticals.

## Conclusion

ITK, which was first discovered in the early 1990s, about 30 years ago, has since then gained more and more attention as a pivotal member and interaction partner in the TCR signalling pathway. In the last 10–15 years, it could be shown in numerous studies that ITK drives inflammatory responses. These can lead to a variety of autoimmune diseases affecting different tissues like lung, gut, skin and even the central nervous system. Moreover, it was demonstrated that patients who have a genetic mutation in the ITK gene locus are more susceptible to virus infection and can thus develop severe diseases affecting the lymphatic system. Especially the oncogenic viruses EBV and HPV were shown to lead to malignant Hodgkin lymphomas in ITK-deficient patients. Consequently, in the recent years, ITK has received attention for the development of inhibitors to tackle these diseases. Although, many different inhibitors have been developed by companies, most of the small molecule inhibitors specifically affecting ITK have not progressed beyond preclinical studies. However, in the European Union, ibrutinib, which is solely marketed as a Btk inhibitor, is approved to treat patients with CLL or mantle cell lymphoma since 2012 and 2013, respectively. Currently, there are new ITK inhibitors in preclinical and clinical trials that need to be further analysed.

In conclusion, over the last 30 years, a great deal of progress has been made from discovering this enzyme to unravelling its structure and molecular function as well as its detrimental role in autoimmune and tumour pathogenesis, placing ITK in the focus as a key target for drug design.

## Abbreviations

APC: antigen-presenting cell
AITL: angioimmunoblastic T cell lymphoma
Bp: base pair
Btk: Bruton’s tyrosine kinase
Ca^2+^: calcium ion
DAG: diacylglycerol
EBV: Epstein-Barr virus
HPV: human papillomavirus
IFNγ: interferon gamma
IL: interleukin
iNKT: invariant natural killer T cell
IP_3_: inositol-1,4,5-triphosphate
ITAM: immunoreceptor tyrosine-based activation motif
ITK: IL-2 inducible tyrosine kinase
ITKKO: IL-2 inducible tyrosine kinase knockout
LP: lamina propria
MHC: major histocompatibility complex
NFAT: nuclear factor of activated T cells
NFκB: nuclear factor ‘kappa-light-chain-enhancer’ of activated B-cells
PH: pleckstrin homology
PI3K: phosphoinositide 3-kinase
PIP_3_: phosphatidylinositol-3,4,5-triphosphate
PLCγ1: phospholipase Cγ1
Rlk: resting lymphocyte kinase
SH: Src homology
TCR: T cell receptor
TFK: Tec family kinases
TH: Tec homolgy
Th: T helper cell
